# miR-34: from bench to bedside

**DOI:** 10.18632/oncotarget.1825

**Published:** 2014-03-15

**Authors:** Massimiliano Agostini, Richard A. Knight

**Affiliations:** ^1^ Medical Research Council, Toxicology Unit, Leicester University, Leicester, UK

**Keywords:** microRNA, miR-34 family, cancer, therapy

## Abstract

The mir-34 family was originally cloned and characterized in 2007 as a p53 target gene. Almost immediately it became clear that its major role is as a master regulator of tumor suppression. Indeed, when overexpressed, it directly and indirectly represses several oncogenes, resulting in an increase of cancer cell death (including cancer stem cells), and in an inhibition of metastasis. Moreover, its expression is deregulated in several human cancers. In 2013, a miR-34 mimic has become the first microRNA to reach phase 1 clinical trials. Here we review the miR-34 family and their role in tumor biology, and discuss the potential therapeutic applications of miR-34a mimic.

## INTRODUCTION

The battle against cancer has just recruited a new potential weapon. Indeed, the first microRNA (miRNA) has now reached phase I studies (http://clinicaltrials.gov/ct2/show/NCT01829971). In April 2013, a study was started to evaluate the safety of MRX34 in patients with unresectable primary liver cancer and advanced or metastatic cancer with liver involvement. The “drug” is given intravenously as a single agent, twice per week for three weeks and then one week off.

miRNAs form one family of small non-coding regulatory RNAs [1]. Several studies have implicated miRNAs in a number of biological processes including cell proliferation, differentiation and the control of developmental timing. They are also involved in pathological conditions such as cancer [2-7] and neurodegeneration [8-11]. The canonical biogenesis of miRNAs involves two fundamental events. The first takes place in the nucleus, where the primary transcript (pri-miRNA), is processed into a precursor (pre-miRNA) by a nuclear RNase III enzyme (DROSHA). The second event occurs in the cytoplasm. The pre-miRNA is exported by exportin V from the nucleus and is cleaved by Dicer into a short-lived dsRNA of about 20-25 nucleotides. This double strand becomes unwound and one strand (forming the mature miR) becomes incorporated into an Argonaut (Ago)-protein containing complex called the RNA induced silencing complex (RISC). Generally, the mature miRNA within the RISC recognises complementary sites in the 3'-UTR of target genes, resulting in translational inhibition or destabilisation of the target mRNAs and downregulation of expression of the encoded protein [12]. Recently, however, some observations have demonstrated that miRNAs can also regulate their targets by binding to the 5'-UTR [13, 14]. In contrast to this classical inhibitory pathway, miRNAs can also stimulate the expression of target genes. This indicates that miRNAs can regulate gene expression not only through base pairing with mRNA targets but also through a decoy activity that interferes with the function of regulatory proteins [15].

The miR-34 family, which consists of miR-34a, b and c, has attracted a lot of attention since it plays a key role as a tumor suppressor in several cancers [16-18]. Indeed, it is a direct target of the tumor suppressor gene p53 [19-23] and when up-regulated, it induces apoptosis [24], cell cycle arrest [25-28] and senescence. It also negatively influences the viability of cancer stem cells and inhibits metastasis formation [29-32]. Moreover, systemic delivery of miR-34 in a mouse model of hepatocellular carcinoma resulted in a reduced tumor burden and prolonged survival [33]. As a result of these and other studies, a miR-34 analogue has become the first microRNA to enter the clinic after a surprisingly swift 6 years passage from the bench to bedside. In addition, since a considerable number of oncogenes are direct targets of miR-34, and cancer is now considered a multipathway disease [34-36], this therapeutic approach would allow the use of only one bullet to hit more than one pathway deregulated by the loss of miR-34.

### The miR-34 Family: origin, regulation and function

The miR-34 gene was first identified in *C. elegans* where it encodes a single miR that is evolutionarily conserved in several invertebrates [37, 38]. In mammals, the miR-34 family consists of three homologous transcripts miR-34a, miR-34b and miR-34c. In man, the miR-34a gene maps to chromosome 1p36.22 and is located within the second exon of its non-coding host gene. It is significant that 1p36 region deletions are frequently observed in a variety of human cancers including neuroblastoma, glioma, breast cancer, non-small cell lung cancer, small cell lung cancer, colorectal cancer and melanoma [39]. However, the genes coding for both miR-34b and miR-34c map to chromosome 11q23.1 and are located within intron 1 and exon 2 respectively, of the same primary transcript. Deletion of this region has been detected in breast, lung, cervical and prostate cancers [40]. Moreover, the 11q23 region is frequently rearranged (translocated, inserted and inverted) in hematological malignancies.[41] In the mouse, miR-34a is located on chromosome 4, while miR-34b/c are located on chromosome 9.

Analysis of miR-34a tissue distribution in the mouse shows that it is ubiquitously expressed but with the highest levels of expression in the brain, while miR-34b/c are mainly expressed in the lung [23], although, in general, the basal expression of miR-34a is higher than that of miR-34b/c. In man, also, miR-34a is ubiquitously expressed with high levels in the ovary, prostate and testes. Intermediate levels are found in brain, lung, thymus and kidney, while liver and heart show low levels of miR-34a. In contrast, miR-34b/c are mainly expressed in the ovary, testes, trachea and lung (http://mirnamap.mbc.nctu.edu.tw).

Although, as mentioned above, the miR-34 family is regulated by p53, it would be more correct to say the p53 family [42]. Retinoic acid induces the expression of miR-34a [43] and we have shown that, at least in the context of terminal differentiation of neuroblastoma cells, is driven by the p53 family member TAp73 [44]. TAp73 is a direct transcriptional activator of miR-34a, since it binds to p53 consensus elements in the miR-34a promoter, but TAp73 does not activate miR-34b and c. This role of the TAp73/miR-34a axis in neuronal differentiation is consistent with the predominantly neuronal phenotype of TAp73 null mice. However, unlike p53, TAp73 activation of miR-34a does not lead to apopotosis – and more work is clearly needed to understand how two members of the p53 family can activate the same miR but with very different biological effects.

**Figure 1:**
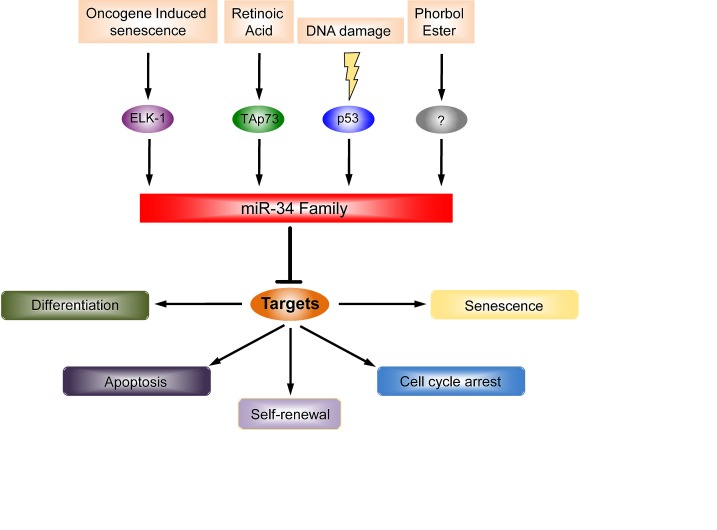
The miR-34 family regulators and their functions In the last few years, the miR-34a family has emerged as a pleiotropic microRNA. It was originally identified as a p53 target after DNA damage. The outcome of this upregulation is the induction of apoptosis, cell cycle arrest and senescence. Lately, p53 independent regulation has been observed. For instance, TAp73 is able to drive the expression of miR-34a and in turn, it regulates neuronal differentiation. miR-34a is also regulated by phorbol ester during megakaryocytic differentiation but the detailed molecular mechanisms have not been defined (?).

Ectopic expression of the members of the miR-34 family can recapitulate some biological functions of p53 such as apoptosis [20, 45] and cell cycle arrest [46, 47], at least in some cell types, although other studies have failed to demonstrate an apoptotic effect of overexpressed miR-34 [44, 48]. Thus, the direct effect on apoptosis by miR-34a, and possibly the absolute requirement for miR-34a for p53-mediated apoptosis, is cell context-dependent. Figure 1 summarizes regulators and functions of the miR-34 family.

Oncogene-induced senescence is another stimulus, which increases expression of miR-34a. Specifically, after constitutive activation of B-RAF in TIG3 fibroblasts, the upregulation of miR-34a is mediated by the transcription factor ELK1 [49]. Thus, like other miRs, the transcriptional regulation of miR-34a expression is promiscuous.

The miR-34 family acts on apoptosis and cell cycle through the repression of many proteins involved in the regulation of these two biological processes. In particular, the miR-34 family binds to the 3'-UTRs of genes such as CDK4 and CDK6 [50, 51] (cell cycle) [19], Bcl-2 [24, 52] (apoptosis), SNAIL [29, 32] (epithelial mesenchymal transition) [53] and CD44 (migration and metastasis) [54], and the miR-34 family thus represses their expression. A detailed list of miR-34 family targets is provided in Table 1.

**Table 1:** miR-34 Targets list in Cancer

Direct Target	miR-34 member	Biological effect	Cancer cell lines/Cancer	Reference
CDK4	miR-34a, b, c	Cell cycle arrest	IMR90, A549, HCT116	19
CDK6	miR-34a, b	Cell cycle arrest	SW480, PC3, Colorectal adenocarcinoma, Prostate cancer, NSCL	23,47, 51
CCNE2	miR-34a, b, c	Cell cycle arrest	SW480, IMR90, A549, HCT116, Lung carcinoma, Colorectal Cancer	19, 23, 63
CCND1	miR-34a	Cell cycle arrest	PC3 A549	47, 51
c-MYC	miR-34b, c	Cell cycle arrest	Burkitt Lymphoma, Raji, Ramos, LCL, SIHN-011B	17, 25
N-MYC	miR-34a	Cell cycle arrest	NLF, IMR32, LAN-5, Neuroblastoma	24, 28
E2F5	miR-34a	Cell cycle arrest	SW480	23
				
CREB	miR-34b	Inhibition of proliferation	K652, HL60, ML2, NB4, NOMO1	92
E2F3	miR-34a, c	Inhibition of proliferation, senescence	PC3, IMR5, SK-N-BE, NLF, HCT116, RKO	17, 24, 43, 48
DLL1	miR-34a	Inhibition of proliferation	Medulloblastoma	100
Notch-2	miR-34a	Inhibition of growth	Glioblastoma	16
YY1	miR-34a	Inhibition of growth	Glioblastoma cells	76
PDGFRA	miR-34a	Inhibition of growth	Glioblastoma cells	27
				
BCL2	miR-34a	Apoptosis	SW480, PC3, NLF, Kato III	23, 24, 52
SIRT1	miR-34a	Apoptosis/p53 activity	PC3, chronic lymphocytic leukaemia	69, 70
Survivin	miR-34a	Apoptosis	Hep-2/ Laryngeal squamous cell carcinoma	18
				
AXIN2	miR-34a	Inhibition EMT	HCT116	30
MET	miR-34a, b, c	Inhibition of invasion and migration	IMR90, A549, HCT116, Lung carcinoma, Colorectal Cancer	19
AXL	miR-34a	Inhibition migration and invasion	NSCLC cell lines, BRC cell lines, CRC cell lines	31
Fra-1	miR-34a/c	Inhibition of invasion and migration	Human primary breast tumors, breast cancer cell lines	94, 95
SNAIL	miR-34a, b, c	Regulation of cancer EMT	H1299, HCT116, SW480, HCT-15	29
CD44	miR-34a	Inhibition of CSC and metastasis	Prostate	54
				
ARHGAP1	miR-34a	Inhibition of invasion and migration	Human lung cancer cells human lung adenocarcinoma	32
				
NANOG	miR-34a	Cancer cell stemness		56
SOX2	miR-34a	Cancer cell stemness		56
Notch-1	miR-34a	Cancer cell stemness	Colon cancer	72
				
LDHA	miR-34a	Glucose metabolism	SW480	86
IMPDH	miR-34a	Purine metabolism	H1299, HCT116	96
HK1	miR-34a	Glucose metabolism	H1299, HCT116	97
HK2	miR-34a	Glucose metabolism	H1299, HCT116	97
GP1	miR-34a	Glucose metabolism	H1299, HCT116	97
PDK1	miR-34a	Glucose metabolism	H1299, HCT116	97
				
SIRT6	miR-34a	Cell differentiation	Squamous cell carcinoma (SCCs)	98
				
WNT1	miR-34a, b, c	Inhibition WNT signalling	MCF-7, A549, SW480, SW620, LoVo, SNU-81	99
WNT3	miR-34a, b, c	Inhibition WNT signalling	MCF-7, A549, SW480, SW620, LoVo, SNU-81	99
LRP6	miR-34a, b, c	Inhibition WNT signalling	MCF-7, A549, SW480, SW620, LoVo, SNU-81	99
β-Catenin	miR-34a, b, c	Inhibition WNT signalling	MCF-7, A549, SW480, SW620, LoVo, SNU-81	99
LEF1	miR-34a	Inhibition WNT signalling	SW480	86
				
MTA2	miR-34a	p53 activity	SW480	86
				

CDK, Cycli-dependent kinase; CCNE, Cyclin E2; CCND1, Cylin D1; CREB, cAMP response element-binding protein; DLL1, Detlta-like protein 1; YY1, transcription factor Ying Yang 1; GP1, glycoprotein 1; PDGFRA,platelet-derived growth factor receptor, alpha polypeptide; BCL2,B-cell lymphoma 2; SIRT1, Sirtuin; AXIN2, Axin-like protein 2; AXL, Tyrosine-Protein Kinase Receptor; HK, hexokinase; Fra-1, Fos-related antigen 1, SNAIL1, Snail Family Zinc Finger 1; ARHGAP1, Rho GTPase Activating Protein 1; NANOG, Homeobox Transcription Factor Nanog; SOX2, SRY(sex determining region Y)-box 2; LDHA, lactate dehydrogenase A; IMPDH, IMP (inosine 5'-monophosphate) dehydrogenase; PDK1, pyruvate dehydrogenase kinase, isozyme 1; WNT, wingless-type MMTV integration site family, member 1; LRP, low density lipoprotein receptor-related protein 6; LEF1, Lymphoid enhancer binding factor 1. EMT, Epithelial–mesenchymal transition; CSC, Cancer stem cells; NSCLC Non-small-cell lung carcinoma; BRC, Breast Cancer; CRC, Colorectal Cancer

The absolute requirement for p53 to drive miR-34 family expression, and for miR-34 family members to mediate the p53 phenotype has, however, recently been questioned. Thus, while, miR-34 expression is reduced in some tissues in p53 null mice, in others it remains unaffected, confirming the promiscuity and cell context dependency referred to above. In particular, miR-34a expression remains high in the brains of p53−/− animals. Moreover, miR-34 knockout mice are born with the normal Mendelian ratio, are fertile, and are not, as might be expected, a phenocopy of the p53 knockout. In particular, miR-34 null mice do not show increased spontaneous or irradiation-induced tumorigenesis, and show only small and subtle differences from wild-type mice in other p53-dependent functions such as replicative senescence and the DNA damage response [55, 56].

### miR34 expression in human cancer

Despite the lack of spontaneous tumours in miR-34 knockout mice, there is evidence, at least in some cancers, for miR-34 dysregulation. Thus, as mentioned above, the miR-34a locus on 1p36 is frequently lost in cancer [57]. Moreover, miR-34a has been found to be downregulated in neuroblastoma [43] and glioblastoma [58, 59], and its expression is frequently reduced in pancreatic cancer cell lines [20, 60]. CpG promoter methylation with miR-34a silencing has also been reported in several cancers including prostate, pancreatic, colorectal, ovarian cancer and melanoma [61, 62]. Mir-34b/c is also downregulated in colorectal cancer (CRC). This down-regulation is associated with hypermethylation of the neighboring CpG island; and DAC (5-aza-2'-deoxycytidine) treatment rapidly restores miR-34b/c expression. Methylation of the miR-34b/c CpG island was frequently observed in CRC cell lines and in primary CRC tumors (101 of 111, 90%), but not in normal colonic mucosa [63, 64].

**Figure 2:**
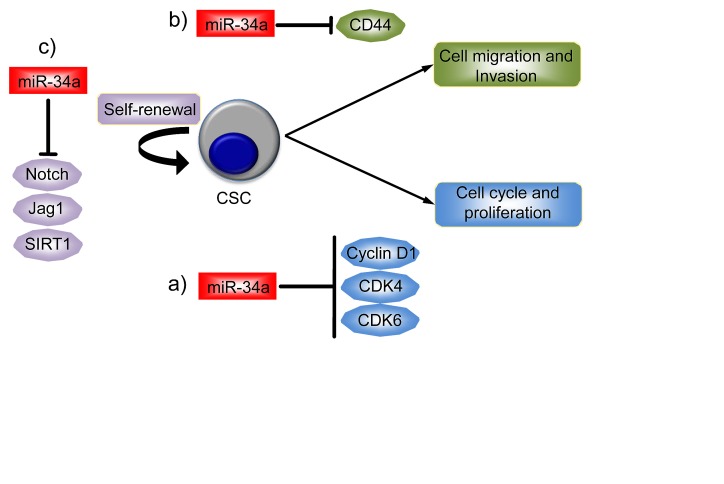
miR-34a as a regulator of cancer stem cell biology Cancer stem cells (CSC) have the capacity to self-renew and differentiate as well as the ability to regenerate tumors. miR-34a has been found to be dysregulated in CSC, particularly in pancreatic, prostatic cancer and in glioblastoma. a) In pancreatic CSC, miR-34a is able to regulate the proliferation of CSC, targeting Cyclin D1, CDK4 and CDK6. b) In prostatic CSC, miR-34a inhibits cell migration and invasion through the inhibition of CD44 expression. c) Finally, in glioblastoma, miR-34a regulates CSC self-renewal through the inhibition of Notch signaling and SIRT1.

There is also some evidence for the involvement of the miR-34 family, again particularly miR-34a, in cancer stem cells (CSCs). CSCs are self-renewing cells within a tumor that have the capacity to regenerate the phenotypic diversity of the original tumor [65-68]. Nalls et al reported the first experimental evidence implicating miR-34a in CSCs [60]. First, they found that the expression of miR-34a was reduced in pancreatic CSCs and in pancreatic tumor cells independently of their p53 status when compared to normal pancreatic ductal epithelial cells. Importantly, the expression of miR-34a was restored by treatment with chromatin modifier agents such as the histone deacetylase inhibitor, Verinostat, in a p53 independent manner. The treatment also inhibited cell growth and induced apoptosis. At the molecular level, the well-known targets of miR-34a (such as SIRT-1 [69-71], Cyclin D1, Bcl-2, VEGF and CDK6) were downregulated and these effects were rescued by miR-34a inhibition (Figure 2a).

**Figure 3:**
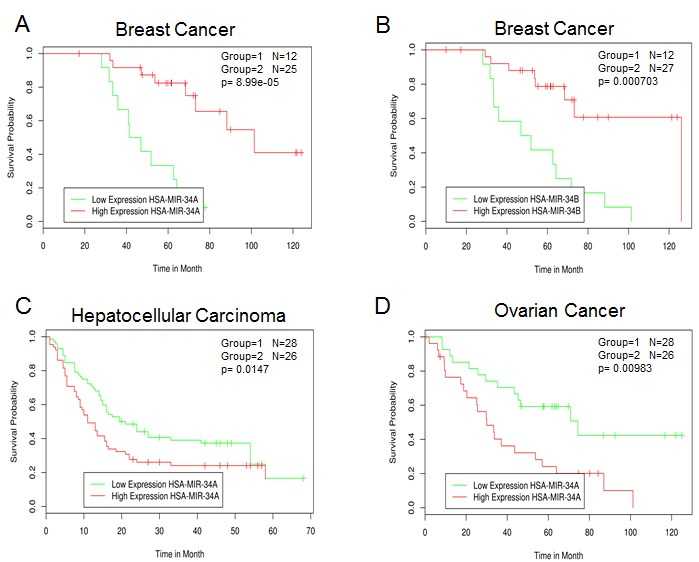
Survival correlation of miR-34 family in several human cancer datasets A and B) GEO dataset Title: Global microRNA expression profiling of high-risk ER+ breast cancers from patients receiving adjuvant Tamoxifen mono-therapy: a DBCG study. C) GEO dataset Title: MicroRNA expression profile in human hepatocellular carcinoma. D) GEO dataset Title: MicroRNA profiling of advanced serous ovarian carcinoma.

Prostate CSCs with tumor initiating and metastatic potential are enriched in the CD44+ subpopulation. In this subset of cells, including CD44+ cells from individual patients tumours, expression of miR-34a, but not miR-34b/c, is also reduced and this does correlate with p53 status [54]. Ectopic expression of miR-34a either in prostate cancer cells or in the CD44+ fraction leads to inhibition of clonogenic expansion, tumor regeneration, and metastasis *in vivo*. In contrast, all these neoplastic phenotypes were promoted when expression of miR-34a was inhibited. Moreover, intravenous delivery of miR-34a inhibited lung metastasis and extended the survival of mice bearing human prostate cancer xenografts. At the molecular level CD44 has been identified and validated as a direct and functional target of miR-34a. Indeed, the inhibition of CD44 expression itself phenocopied miR-34a overexpression by inhibiting prostate tumor development and metastasis (Figure 3b).

miR-34a expression is also reduced in human glioblastoma tissue when compared with normal brain [16], although this downregulation of miR-34a was only seen in tumours with mutant p53 and not in glioblastomas with wild-type p53. Transfection of precursor miR-34a in glioblastoma cells, as well as in glioblastoma CSCs, induced cell cycle arrest, apoptosis and also inhibited xenograft growth. The effects of miR-34a on glioma cells are partially mediated by the inhibition of c-Met and Notch expression, and levels of miR-34a are inversely correlated with the levels of c-Met in human gliomas. A role of miR-34a has been also shown in colon cancer stem cells (CCSCs) [72]. In this cellular context, miR-34a controls the decision of CCSCs to perform either symmetric or asymmetric division. Mechanistically, high levels of miR-34a reduce Notch1 signaling and promote asymmetric division. In contrast, low miR-34a levels upregulate Notch1 signaling and promote symmetric division. Promoting cell differentiation is another mechanism by which miR-34a exerts its tumor suppressor function in CRC (Figure 3c).

### miR-34 family survival analysis in cancer

Overall, miR-34a and its family are tumor suppressors. Therefore, we would predict that the reduction of miR-34 expression is associated with poor prognosis and survival. Using MIRUMIR [73, 74] (http://www.bioprofiling.de/GEO/MIRUMIR/mirumir.html), an online tool that provides an analysis of miRs as potential biomarkers to predict survival of cancer patients, the following picture has emerged. Several datasets (breast, prostate and lung cancer, ovarian, hepatocellular and nasopharyngeal carcinoma) are currently available although statistical significance was only reached in three of them. Low expression of miR-34a/b was associated with poor outcome in breast cancer (Figure 3A and 3B) confirming its role as tumor suppressor. Moreover, the expression of several miR-34a/b validated targets including BCL-2 [75], CCNE2 [52], CCND1 [50], E2F3 [24, 43], MET [19], CD44 [54] and YY1 [76, 77] correlates with survival across different breast cancer datasets. In contrast, low levels of miR-34a are positive prognostic factors in human hepatocellular and advanced serous ovarian carcinoma (Figure 3C and 3D). Although this is at first sight surprising, this correlation analysis of the human hepatocellular carcinoma dataset is in agreement with a previous report, and may be further evidence for the cell context dependency of the biological effects of miR-34. Indeed, Pineau et al observed that miR-34a expression was increased in hepatocellular carcinoma and was linked to disease progression from normal liver through cirrhosis to full-blown hepatocellular carcinoma [78]. In human ovarian cancer (83 samples) miR-34 family expression was found to be reduced when compared to six (apparently mouse) ovarian surface epithelium cell samples. However, there were no significant differences when the expression of miR-34a was compared between stage III and stage IV distant metastatic disease [79]. Clearly, future studies are required in order to have a more coherent picture of the miR-34 family regulation in cancer and whether this family can be used as a prognostic biomarker [80, 81].

### Perspectives and Conclusions

The last 7 years of studies have clearly shown that the miR-34 family is a master regulator of tumor biology. The family is frequently deregulated in cancer as discussed above, and preclinical *in vivo* studies have highlighted its therapeutic potential [82-84]. Overall, miR-34a (or a mimic) would seem a perfect candidate to enter clinical trials. However, it should be remembered that miR-34 and p53 have independent functions. Thus, miR34 null mice do not develop spontaneous tumors like p53 knockout mice [55]. In addition, the correlation between miR-34 family expression and patient survival would not always support its tumor suppressor role. In conclusion, more preclinical research on miR-34 is needed in order to better characterize its regulation and its downstream molecular pathways.

On the other hand, the phase 1 clinical trail that has recently started represents an important step forward not only for miR-34 itself, but forms a valuable proof of principle study for the rationale of using miRNAs as anticancer drugs. Indeed, although the endpoint of this clinical trial at this stage is to investigate the safety, pharmacokinetics and pharmacodynamics of the miR-34 mimetic in patients with unresectable primary liver cancer, it might shed light on two main challenges for miRNA-based therapies: i) delivery system and ii) potential off-target effects.

Tissues-specific delivery and cellular uptake of sufficient amounts of synthetic oligonucleotides to achieve sustained target inhibition is one of the major issues. Indeed, in miRNA-based therapy, two relevant obstacles need to be overcome, including the biological instability of the oligonucleotides in tissues and the poor cellular uptake [85]. MRX34 is a double stranded RNA, which is delivered by liposome. Since liposomes accumulate in the liver, liver cancer would theoretically be the main target organ affected by MRX34. Moreover, tumor uptake should be enhanced by the particular chemical composition of the liposomes. These liposomes are anionic at normal body pH, but in the tumor microenvironment, which has a lower pH, they become cationic form. This characteristic should therefore provide tumor specificity and prevent uptake by normal tissues.

One of the main advantages of miRNA-based therapy is the fact that microRNAs have the ability to simultaneously regulate several cellular pathways [86-89]. This makes them suitable “drugs” for the treatment of a multipathway disease such as cancer [90, 91]. In contrast, this multi-target property of the microRNA could potentially result in off-target side effects. Systemic overexpression of miR-34, which is broadly expressed and regulates physiological processes, could target genes in healthy tissues and cause side effects such as cardiovascular disease, although this may be minimised by the use of the particular liposome formulation [92].

In conclusion, while we should celebrate the entry of miR therapy into the oncologists drug cupboard, we should use the opportunity to learn as much about the disadvantages and qualifications of this new approach in order to optimize its therapeutic application in the future.
